# 15-Deoxy-Δ^12,14^-Prostaglandin J_2_ Modifies Components of the Proteasome and Inhibits Inflammatory Responses in Human Endothelial Cells

**DOI:** 10.3389/fimmu.2016.00459

**Published:** 2016-10-27

**Authors:** Simone Marcone, Paul Evans, Desmond J. Fitzgerald

**Affiliations:** ^1^UCD Conway Institute, School of Medicine and Medical Science, University College Dublin, Dublin, Ireland; ^2^Centre for Synthesis and Chemical Biology, School of Chemistry, University College Dublin, Dublin, Ireland

**Keywords:** 15d-PGJ_2_, proteasome, covalent modification, inflammation, adhesion molecules, chemokines, monocyte adhesion, atherosclerosis

## Abstract

15-Deoxy-Δ^12,14^-prostaglandin J_2_ (15d-PGJ_2_) is an electrophilic lipid mediator derived from PGD_2_ with potent anti-inflammatory effects. These are likely to be due to the covalent modification of cellular proteins, *via* a reactive α,β-unsaturated carbonyl group in its cyclopentenone ring. This study was carried out to identify novel cellular target(s) for covalent modification by 15d-PGJ_2_ and to investigate the anti-inflammatory effects of the prostaglandin on endothelial cells (EC). The data presented here show that 15d-PGJ_2_ modifies and inhibits components of the proteasome and consequently inhibits the activation of the NF-κB pathway in response to TNF-α. This, in turn, inhibits the adhesion and migration of monocytes toward activated EC, by reducing the expression of adhesion molecules and chemokines in the EC. The effects are consistent with the covalent modification of 13 proteins in the 19S particle of the proteasome identified by mass spectrometry and the suppression of proteasome function, and were similar to the effects seen with a known proteasome inhibitor (MG132). The ubiquitin–proteasome system has been implicated in the regulation of several inflammatory processes and the observation that 15d-PGJ_2_ profoundly affects the proteasome functions in human EC suggests that 15d-PGJ_2_ may regulate the progression of inflammatory disorders such as atherosclerosis.

## Introduction

Prostaglandins are a family of related compounds derived from arachidonic acid that are released from cell membrane stores in response to a range of stimuli including cytokines (1). One of these products, PGD_2_, is an abundant prostaglandin in several tissues. PGD_2_ activates G-protein-coupled receptors on its target cells, but PGD_2_ also undergoes dehydration *in vivo* and *in vitro* to generate cyclopentenone metabolites of the J series, including 15-deoxy-delta^12,14^-prostaglandin J_2_ (15d-PGJ_2_) (2, 3). 15d-PGJ_2_ represses inflammatory responses in several models, including modulation of genes such as iNOS, TNF-α, and COX-2 (4, 5). 15d-PGJ_2_ was also identified as a potent ligand of the nuclear receptor, PPAR-γ (6–8). There is evidence that at least some of these anti-inflammatory effects are mediated through the covalent modification of cellular proteins, *via* its reactive α,β-unsaturated carbonyl group (9–15), which in turn modifies their biological functions (14).

One key target of 15d-PGJ_2_ is the NF-κB pathway comprising a cluster of proteins that regulate the inflammatory responses in cells. The NF-κB family consists of RelA (p65), NF-κB1 (p50/p105), NF-κB2 (p52/p100), c-Rel, and RelB. The p50 and p52 subunits are derived on proteolytic cleavage of precursors p105 and p100, respectively, by the proteasome. The binding of Rel proteins to p50 and p52 forms dimeric complexes that in turn bind DNA to regulate the transcription of many inflammatory genes, including cytokines, chemokines, and adhesion molecules, as well as antiapoptotic and antioxidant genes. In many cell types, the most abundant form of NF-κB is the p50/p65 heterodimer, which binds to a responsive element found in the promoter of several atherogenic genes, including adhesion molecules and chemokines which, in turn, regulate monocyte recruitment (16). The NF-κB heterodimer remains in an inactive form in the cytoplasm, forming a complex with the inhibitory protein of NF-κB, the IκBs. Potent NF-κB activators, such as TNF-α, cause almost complete degradation of IkBs (especially IkB-α) within minutes. This process is mediated by the 26S proteasome and depends on phosphorylation of IkBs. The control of IkB phosphorylation is mediated by IkB kinase (IKK) complex. 15d-PGJ_2_ inhibits IKK, thus preventing IkB degradation and NF-κB nuclear translocation (17). It can also directly modify NF-κB subunits blocking their ability to bind DNA (18). Activation of the NF-κB pathway is further dependent on the function of the ubiquitin–proteasome system (UPS), which is a key regulator of the protein turnover and degradation in human cells. Inflammatory cell signaling promotes the dissociation of IκB-α from NF-κB and is processed by the proteasome. Inflammatory cell signaling also promotes the processing of the p105 precursor by the proteasome, to generate mature P50 subunits. If the proteasome is inhibited, degradation of both IκB-α and p105 is prevented and they remain complexed to NF-κB heterodimer preventing its activation (19).

The 26S proteasome is a large proteolytic complex that regulates a variety of important physiologic and pathologic cellular processes by selective degradation of proteins (20). The 26S complex consists of two asymmetric 19S caps linked to a barrel-shaped core, the 20S proteasome. During the process of degradation, a polyubiquitinated protein is first recognized by the 19S regulatory particle, unfolding the protein and translocating it to the 20S core particle where it is subjected to various types of protease activity (21). The UPS regulates cellular processes and pathways implicated in the development of many diseases. For example, proteolysis and protein turnover in the brain are key processes in the formation of protein deposits in neurodegenerative disease (22). Also in cancer, UPS has been shown to control the abundance and activity of oncogenes and to promote tumorigenesis directly by the degradation of tumor suppressor p53 (23). Thus, proteasome inhibitors are currently used in cancer therapy (24).

Other inflammatory diseases have been shown to involve UPS, including atherosclerosis (25), in which the proteasome activity has been linked to foam cell formation, and to the smooth muscle cell transformation, proliferation, and migration that are characteristics of atherosclerotic plaque (26). In the current work, we explored whether inhibition of proteasomal activity occurred as a consequence of covalent modification of its component proteins by 15d-PGJ_2_, and the effect of 15d-PGJ_2_ on UPS activity, and the inflammatory responses, of human endothelial cells (EC).

## Materials and Methods

### Antibodies

NF-κB p65, p50, and p105 subunits antibodies and Ik-β and anti-rabbit secondary antibody were obtained from Cell Signaling Technology (MA, USA). The β-actin, anti-ubiquitin, anti-biotin, PSMD2, PSMD3, PSMD11, 20S Proteasome β1, 20S Proteasome α1, polyclonal anti-mouse secondary antibodies, and Protein A/G plus agarose beads were from Santa Cruz Biotechnology (CA, USA). Anti-PSMD1 and anti-TBP antibodies were from Sigma-Aldrich (Dorset, UK).

### Cell Culture and Prostaglandin Treatments

Human aortic endothelial cells were obtained from Cascade Biologics – Invitrogen cell culture (Carlsbad, CA, USA) and were grown in EC culture media MV plus growth supplements from Promo Cell (Heidelberg, DE, USA) supplemented with 100 units/ml penicillin and 100 μg/ml streptomycin. For experiments, 80% confluent ECs were incubated in EC basal media from Promo Cell (2% fetal calf serum, 0.4% EC growth supplements, 90 μg/ml heparin and with 100 U/ml penicillin, and 100 μg/ml streptomycin) for 24 h before treatments. THP-1 monocytes were purchased from the LGC Promochem (Middlesex, UK) and were maintained in RPMI 1640 media supplemented with 10% fetal bovine serum, 1% l-glut, and 1% P/S. The cells were passaged twice weekly in complete media at a ratio of 1:5. All cell lines were maintained in an incubator at 37°C and 5% CO_2_. For cell counts and viability determination, the cells were diluted 1:1 with trypan blue and counted using a hemocytometer. 15-Deoxy-Δ^12,14^-prostaglandin J_2_, biotin-15-deoxy-Δ^12,14^-prostaglandin J_2_, prostaglandin D_2_, and biotin-prostaglandin D_2_ were purchased from Cayman Chemical (Ann Arbor, MI, USA). To change the solvent for experiments, prostaglandins were dried down and re-suspended in ethanol at a concentration of 1 mg/ml. For experiments, ethanol was used as vehicle control.

### Immunoprecipitation

Endothelial cells were treated with prostaglandins for 4 h at 37°C. After incubation, the EC were solubilized in lysis buffer (1% Non-idet P-40, 0.1% SDS, 150 mM NaCl, 50 mM Tris–HCl, pH 7.2) and complete protease inhibitor (Roche Diagnostics, Germany). Immunoprecipitation of human plaques proteins was carried out by solubilizing the tissues in T-PER Tissue Protein Extraction Reagent (Thermo Scientific, IL, USA) and complete protease inhibitor (Roche Diagnostics, Germany) and subsequent homogenization in a tissue lyser. The samples were centrifuged (12,000 × *g* for 10 min at 4°C) and 300 μg of total cell extract was diluted to 500 μl with lysis buffer. The sample was incubated in 2 μg of antibody at 4°C for 2 h and 25 μl of Protein A/G plus agarose beads added to the sample prior to incubation overnight at 4°C. The immunoprecipitates were washed four times with lysis buffer and proteins were eluted with SDS sample buffer (63 mM Tris–HCl, 10% glycerol, 2% SDS, 0.0025% bromophenol blue, pH 6.8) and analyzed by SDS-PAGE, followed by Western blotting. Antibodies to PSMD2, PSMD3, PSMD11, 20S Proteasome α1, and 20S Proteasome β1 were obtained from Santa Cruz Biotechnology (CA, USA) and used for immunoprecipitation experiments.

### SDS-PAGE and Western Blotting

Protein samples of interest were run on 4–20% polyacrylamide gels and subjected to electrophoresis. The gels were electrophoresed in Tris–HEPES running buffer (0.1M Tris, 0.1M HEPES, 3 mM SDS, pH 8) using a Mini-PROTEAN 3 Electrophoresis System (Biorad) at 80 V for 15 min and 120 V for the duration of the run. SDS-PAGE separated proteins were transferred to nitrocellulose membrane in transfer buffer (25 mM Tris, 192 mM glycine, 20% v/v MeOH). Transfer was carried out for 90 min at 80 V on ice. The membranes were stained with the reversible stain Ponceau-S solution to check transfer efficiency and destained in distilled water. The membranes ware then blocked for 2 h in 5% BSA in TBS-T (20 mm Tris, pH 7.6, 150 mm NaCl, 0.1% Tween 20) at room temperature on an orbital shaker. The membranes were subsequently incubated with the appropriate dilution of primary antibody overnight. Following incubation, the membranes were washed three times in TBS-T and incubated with the appropriate dilution of secondary antibody for 1.5 h. After three washes in TBS-T for 15 min, the membranes were developed using Supersignal enhanced Chemiluminesence reagents (Thermo Scientific, IL, USA) and exposed to photographic X-ray film.

### Bioinformatic Analysis

The 358 proteins covalently modified by 15d-PGJ_2_, in EC (27), were subjected to pathway mapping analysis and were distributed into categories according to their cellular component, molecular function, and biological process using Gene Ontology (GO), a web-based platform available for interpreting set of GO hierarchies and the PANTHER classification system.^1^ The identified proteins were subjected to pathway mapping analysis using the KEGG pathway database (DAVID Bioinformatics Resources 6.7). To filter the results, a threshold of two proteins per pathway and a significance level of *p* < 0.01 were applied.

### MTT Assay for Cell Viability

The cell viability of EC treated with 15d-PGJ_2_ or MG132 was evaluated by MMT assay. The following dose–response was applied for 15d-PGJ_2_: 0, 1, 5, 10, and 25 μM. The cell viability after treatment with MG132 was assayed at 100 nM (the dose used for experiments). After 18 h of treatment, 5 mg/ml of dimethylthiazol-diphenyltetrazolium bromide (MTT) (Sigma-Aldrich, Ireland) solution was added to EC and incubated at 37°C, 5%CO_2_ for 3.5 h. Following cell lysis with DMSO, absorbance was spectrophotometrically read at 570 nm. For experiments, 15d-PGJ_2_ doses which did not significantly reduce cell viability were chosen (97.2 ± 4% viability with 1 μM 15d-PGJ_2_, 95.4 ± 4% with 5 μM, and 90.5 ± 6% with 10 μM), while EC viability was significantly decreased at 72.7 ± 8% with 25 μM 15d-PGJ_2_. EC viability treated with 100 nM MG132 was 93.5 ± 4%.

### Proteasome Activity Assay

Lysates of EC treated with 15d-PGJ_2_ or MG132 for 18 h were used to measure the chymotrypsin-like activity of the proteasome using the peptide substrate SUC-LLVY-AMC and 50 μg of proteins per assay. A commercially available kit (Cayman Chemical, Ann Arbor, MI, USA) was used to perform the assay according to the manufacturers’ guidelines. AMC hydrolysis was measure in a Spectramax M2 (Molecular Devices, CA, USA) plate fluorescence reader with 360-nm excitation and 460-nm emission wavelength. The proteasome inhibitor epigallocatechin gallate (EGCG) was used as control to demonstrate specificity of the substrate in this assay. The experiments were repeated in triplicate.

### 1 9S Proteasome-15d-PGJ_2_-Binding Assay

A high-binding polystyrene microplate (R&D, MN, USA) was coated overnight at 4°C with 40 μg/ml of purified 19S Proteasome (Boston Biochem, MA, USA) in PBS. After incubation, the plate was washed four times with 0.05% Tween 20 in PBS and 100 μl solution of biotinylated-15d-PGJ_2_ or biotinylated-15d-PGD_2_ in PBS was added to the wells and incubated for 2 h at RT with gentle shaking. A binding competition assay was performed to determine the binding specificity of biotinylated-15d-PGJ_2_ to the proteasome proteins. Specifically, the 19S-coated plate was pre-incubated with 10- and 20-fold molar excess of 15d-PGJ_2_ for 2 h at RT and subsequently with biotinylated-15d-PGJ_2_ for a further 2 h at RT. After incubation, the plate was washed four times with 0.05% Tween 20 in PBS. After washing, a blocking solution of 4% BSA in PBS was subsequently added for 2 h at RT with gentle shaking. Subsequently, a solution of 1:750 (v:v) streptavidin–horseradish peroxidase conjugate (GE Healthcare, USA) was incubated for 1 h at RT. After washing four times with 0.05% Tween 20 in PBS, 100 μl of substrate reagent solution (R&D, MN, USA) was incubated for 30 min at RT with gentle shaking. The reaction was stopped with 25 μl of 2M sulfuric acid, and the absorbance was read at 450 nm with correction at 540 nm in a Spectramax M2 (Molecular Devices, CA, USA) plate reader.

### Immunocytochemistry

Endothelial cells were grown on cover slips for fluorescence microscopy and treated with 15d-PGJ_2_ or MG132 for 18 h and subsequently stimulated with 0.2 ng/ml TNF-α for 30 min. The cells were fixed with formaldehyde (3%) for 15 min followed by blocking in 5%BSA in PBS for 1 h. EC were then incubated with the appropriate dilution of primary antibody followed by incubation with fluorescent secondary antibody (Alexa Fluor 488, Invitrogen). Subsequently, the cells were stained with Alexa Fluor 568-phalloidin to stain actin microfilaments and with DAPI to visualize nucleic acid. The stained cells were analyzed by using a Zeiss AxioImager M1 fluorescent microscope, and the images were captured using an Olympus digital camera (Optronics, Goleta, CA, USA). Pictures were processed using AxioVision software.

### Adhesion Assay

For static adhesion assay, 4 × 10^4^ EC were seeded in a 96-well plate for 24 h and then treated with 15d-PGJ_2_ or MG132, for 18 h. Subsequently, EC were stimulated with TNF-α (0.2 ng/ml) for 6 h. After treatments, the wells were washed three times with medium, and 1 × 10^5^ fluorescein-labeled THP-1 monocytes were added to each well and incubated for 30 min at 37°C. After incubation, the wells were washed three times with fresh medium and adherent monocytes were measured in a Spectramax M2 (Molecular Devices, CA, USA) plate fluorescence reader with 485-nm excitation and 530-nm emission wavelength. For photomicrographs, fluorescence-labeled adherent monocytes in the 96-well plate were analyzed using a Zeiss AxioImager M1 fluorescent microscope. Adhesion assay experiments were repeated in triplicate, and the average value was expressed as a percentage of vehicle control.

### Migration Assay

THP-1 monocytes were applied to 24-well transwell migration plates (Invitrogen, USA), consisting of upper and lower chambers, separated by membranes punctuated with pores of 5 μm in diameter. The 1 × 10^5^ monocytes were seeded in a final volume of 150-μl basal medium into the upper chamber. Conditioned medium was prepared from confluent EC treated with 15d-PGJ_2_ or MG132 for 18 h; the cells were washed, fresh medium was added, and then the cells were treated with TNF- α (0.2 ng/ml for 6 h). After incubation, conditioned medium was cleared by centrifugation (14,000 × *g* for 10 min at 4°C). Conditioned medium was added to the lower chamber in a final volume of 500 μl as a chemoattractant. Plates were incubated for 2 h at 37°C. Membranes were washed with PBS and were placed into 4% formaldehyde for 15 min to fix cells adherent to the underside of the membrane. Membranes were washed with PBS, and the upper chambers immersed in DAPI solution for 3 min, for nucleic acid staining. Following two PBS washes, the adherent cells were visualized using a Zeiss AxioImager M1 fluorescent microscope. The number of cells in five random 20× fields was counted, and the average value was expressed as a percentage of control.

### Flow Cytometry

Following 15d-PGJ_2_ or MG132 treatments (18 h) and activation with TNF- α (0.2 ng/ml for 6 h), the EC were harvested to be stained for flow cytometric analysis. To block non-specific binding, EC were incubated in 2% BSA in PBS (blocking buffer) for 15 min before adding the antibodies. FITC-VCAM-1, PE-ICAM-1, and APC-E-selectin antibodies (BD, Rankling Lakes, NJ, USA) were used to label EC in 2% BSA in PBS. The antibodies were incubated for 30 min at room temperature. Following two washes with blocking buffer, the cells were fixed in 2% paraformaldehyde. Forward and side scatter gates were established to exclude non-viable cells and cell debris from the analysis. The mean fluorescence intensity of 2 × 10^5^ cells was analyzed in each sample. Auto-fluorescence signals generated by unlabeled cells were used as negative controls in each experiment. Flow cytometric analysis was performed on an Accuri C6 instrument and analyzed with CFlow^®^ Software (Accuri, Ann Arbor, MI, USA). The data were expressed as median fluorescence intensity of three independent experiments.

### Statistical Analysis

The data were analyzed by parametric unpaired Student’s *t*-test and analysis of variance (ANOVA). The Student’s *t*-test was used to compare the mean of two data sets. ANOVA was used to examine any overall differences between treatments groups. Results were expressed as mean ± SEM. Experimental points were performed in triplicate with a minimum of three independent experiments. A value of *p* < 0.05 was considered statistically significant.

## Results

### The Proteasome Complex Is a Target of 15d-PGJ_2_

**Figure 1:**
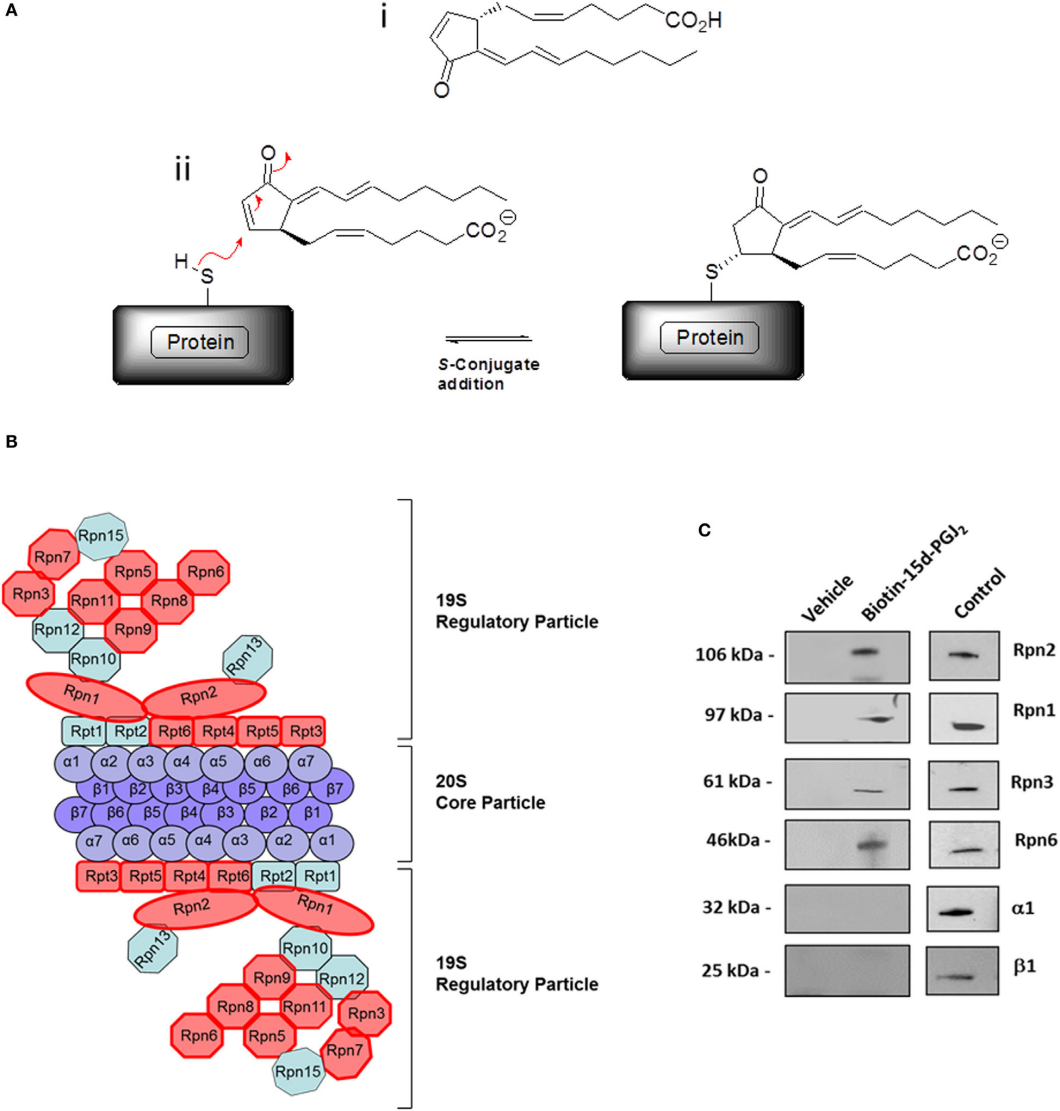
**The proteasome complex is a target of 15d-PGJ_2_**. **(A)** (i) Structure of 15d-PGJ_2_ and (ii) mechanism of covalent modification of thiol-containing proteins by 15d-PGJ_2_ *via* S-conjugate addition. **(B)** EC were incubated with 5 μM biotin-15d-PGJ_2_ and biotin-15d-PGJ_2_ modified proteins were bound to neutravidin beads, analyzed by SDS-PAGE, and identified by LC–MS/MS (27). The red shapes in the schematic representation indicate the 13 proteins in the 19S regulatory particle modified by 15d-PGJ_2_. **(C)** Validation of MS results by Western blotting. EC were treated with or without 5-μM biotin-15d-PGJ_2_, and the proteasome proteins were affinity-precipitated using anti Rpn1, Rpn2, Rpn3, and Rpn6 antibodies. Modification by biotin-15d-PGJ_2_ was demonstrated by anti-biotin Western blot. Total cell lysates were used as positive controls (Control) for each antibody. Anti-20S proteasome antibodies (α1 and β1) were used to show the localization of the 15d-PGJ_2_ modification to the 19S regulatory subunits of the 26S proteasome.

In our previous work (27), conjugation targets of 15d-PGJ_2_ were identified by a chemical proteomic approach where human EC were exposed to 5 μM 15d-PGJ_2_ that had been biotinylated at the carboxyl group, leaving the reactive cyclopentenone ring unmodified. A total of 358 proteins complexing with 15d-PGJ_2_ were identified by affinity purification and subsequent liquid chromatography–tandem mass spectrometry (LC–MS/MS) (27). The MS data are available in PRIDE proteomics identification database^2^ under accession numbers 27957–27962. Functional annotations were available for 355 of the 358 proteins identified. The analysis showed the proteasome complex to be disproportionally represented (fold enrichment = 8.30, *p* = 2.20E−08) with 13 proteins in this pathway identified in our dataset. The identified proteasome proteins, together with the corresponding MS identification details, are detailed in Table 1. Validation of several 15d-PGJ_2_ targeted proteasome proteins (Rpn1, Rpn2, Rpn3, and Rpn6) was performed by immunoprecipitation from EC treated with 5 μM biotin-15d-PGJ_2_ and subsequent anti-biotin Western blot. As shown in Figures 1B,C, only the 19S proteasome proteins were modified by biotin-15d-PGJ_2_, while proteins in the 20S proteasome (α1 and β1) were not modified. These results show an extraordinary level of specificity for the interaction of the proteasome with 15d-PGJ_2_.

**Table 1:** **List and details of proteasome proteins identified by liquid chromatography–tandem mass spectrometry (LC–MS/MS)**.

UniProt ID	Protein identification	MASCOT score	Sequence coverage	pI	Peptide hits
PSMD2	26S proteasome non-ATPase regulatory subunit RPN1	107	39.4	5.08	21
PSD12	26S proteasome non-ATPase regulatory subunit RPN5	94	39.3	7.53	21
PSD11	26S proteasome non-ATPase regulatory subunit RPN6	94	55.7	6.08	19
PRS8	26S protease regulatory subunit RPT6	87	45.6	7.11	18
PRS6B	26S protease regulatory subunit RPT3	72	41.6	5.09	8
PSD7	26S proteasome non-ATPase regulatory subunit RPN8	71	22.2	6.29	5
PRS6A	26S protease regulatory subunit RPT5	70	30.3	5.13	13
PSMD6	26S proteasome non-ATPase regulatory subunit RPN7	65	40.1	5.45	14
PSMD3	26S proteasome non-ATPase regulatory subunit RPN3	53	32.2	8.47	13
PSD13	26S proteasome non-ATPase regulatory subunit RPN9	50	27.9	5.53	9
PSDE	26S proteasome non-ATPase regulatory subunit RPN11	49	35.5	6.06	7
PSMD1	26S proteasome non-ATPase regulatory subunit RPN2	43	33.1	5.25	24
PRS10	26S protease regulatory subunit RPT4	42	24.4	7.1	7

*Biotin-15d-PGJ_2_-modified proteins from EC treated with 5 μM biotin-15d-PGJ_2_ were purified by affinity precipitation using neutravidin beads, separated by SDS-PAGE, and identified by LC–MS/MS*.

### 15d-PGJ_2_ Covalently Binds to 19S Proteasome Proteins, Inhibits Proteasome Activity, and Induces Accumulation of Proteins Conjugated to Ubiquitin

**Figure 2:**
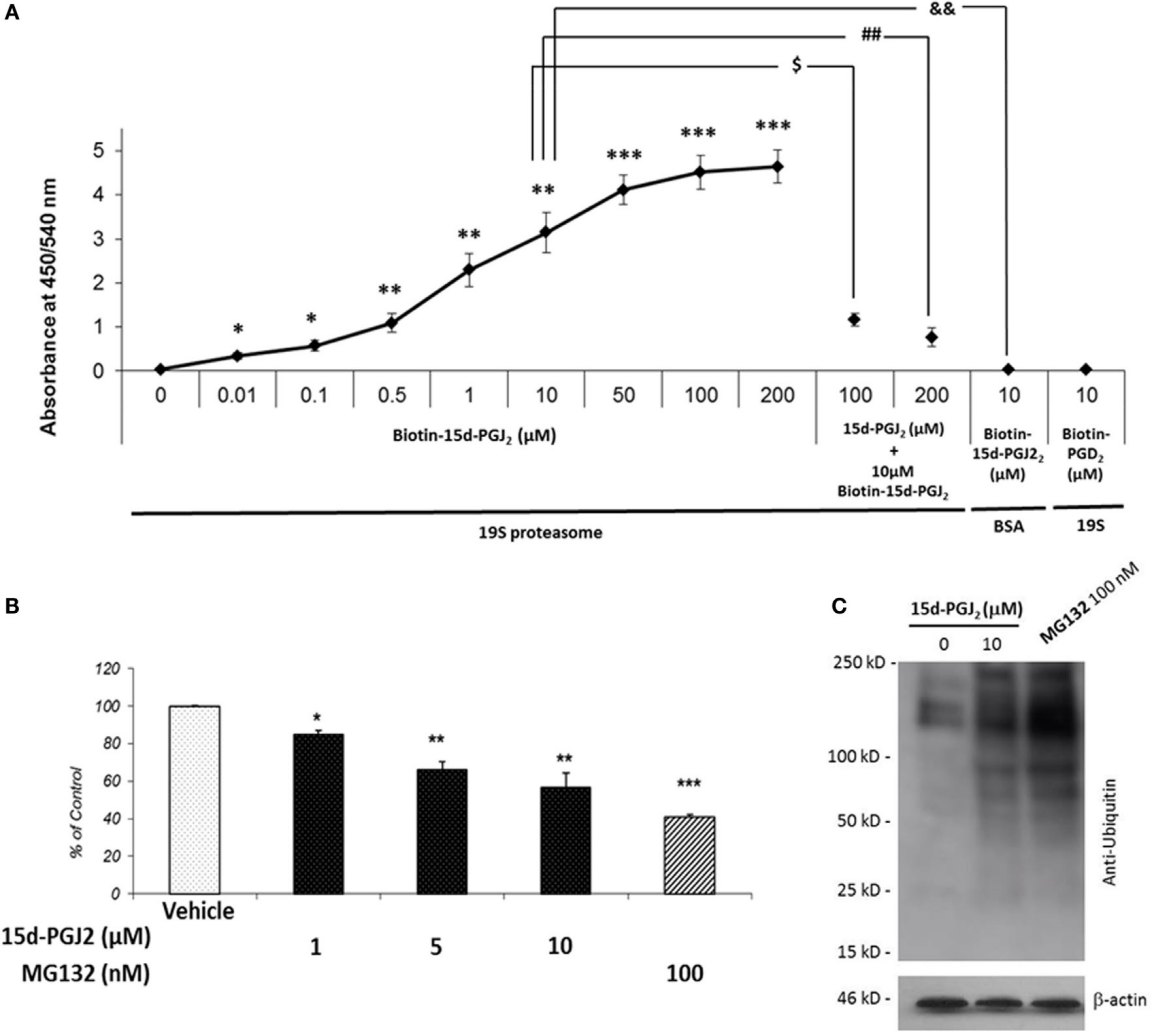
**15d-PGJ_2_ covalently binds to 19S proteasome proteins, inhibits proteasome activity, and induces accumulation of proteins conjugated to ubiquitin**. **(A)** The interaction of biotin-15d-PGJ_2_ with purified 19S proteasome was assayed on a 19S-coated ELISA plate. The specificity of the interaction was confirmed in competition assays using 10- and 20-fold molar excess of unlabeled 15d-PGJ_2_, and by using biotin-PGD_2_. Data are expressed as mean ± SEM vs. control of three independent experiments. ***p < 0.001, **p < 0.01, and *p < 0.05 biotin-15d-PGJ_2_ vs. vehicle (0 μM biotin-15d-PGJ_2_). ^$^(*p* < 0.05) 10-fold molar excess of unlabeled 15d-PGJ_2_ vs. biotin-15d-PGJ_2_; ^##^(*p* < 0.01) 20-fold molar excess of unlabeled 15d-PGJ_2_ vs. biotin-15d-PGJ_2_; ^&&^(*p* < 0.01) biotin-15d-PGJ_2_ in the absence of 19S proteasome. **(B)** The peptidase activity of the proteasome in EC lysates treated with 15d-PGJ_2_ for 18 h was measured using a fluorogenic substrate (SUC-LLVY-AMC). For comparison, the effect of the known proteasome inhibitor MG132 is shown. Inhibition of proteasome activity was expressed as fluorescence unit per wavelength and reported as percentage of control (ethanol) (mean ± SEM, *n* = 3); ***p < 0.001, **p < 0.01, and *p < 0.05 15d-PGJ_2_ or MG132 vs. vehicle. **(C)** Anti-ubiquitin Western blot analysis of EC treated with 10 μM 15d-PGJ_2_ or with 100-nM MG132 for 18 h resulted in accumulation of proteins conjugated to ubiquitin, consistent with a decreased proteasomal activity.

To further examine the covalent modification of 19S proteasome proteins by 15d-PGJ_2_, an immunoplate was coated with purified 19S particle of the 26S proteasome and incubated *in vitro* with biotin-15d-PGJ_2_. Biotin labeling of 19S proteasome was observed at nanomolar concentration of biotin-15d-PGJ_2_ (Figure 2A) demonstrating direct interaction of 15d-PGJ_2_ and proteasome proteins at low levels of the prostaglandin. The specificity of the interaction was confirmed by pre-incubating the coated plate with 10- and 20-fold molar excess of unlabeled 15d-PGJ_2_, which prevented the binding of biotinylated prostaglandin, and by incubating 10 μM biotin-15d-PGJ_2_ with an irrelevant protein, BSA. In addition, biotin-PGD_2_ showed no binding, confirming the specificity of the interaction between 15d-PGJ_2_ and the 19S. In our previous work, we demonstrated that covalent modification by 15d-PGJ_2_ profoundly altered the biological function of several proteins (14). Here, we explored the effect of 15d-PGJ_2_ covalent modification of proteasome components on proteasome catalytic activity and assayed in lysates of EC pretreated with the prostaglandin. The effects were compared to that of MG132, a well-known synthetic proteasome inhibitor which acts by covalently binding to the beta subunits of the proteasome (28). The proteolytic activity of the proteasome was assayed using a fluorogenic substrate (SUC-LLVY-AMC) (Figure 2B). The treatment with 15d-PGJ_2_ inhibited the proteasome activity by 16 ± 2% at 1 μM, 36 ± 4% at 5 μM, and 44 ± 7% at 10 μM compared to the vehicle control. For comparison, 100-nM MG132 decreased the proteasome activity by 58 ± 1%. The proteasome system degrades proteins that have been targeted for proteolysis by the addition of ubiquitin molecules. As shown in Figure 2C, anti-ubiquitin Western blot analysis of EC treated with 10-μM 15d-PGJ_2_ or with 100-nM MG132 resulted in accumulation of proteins conjugated to ubiquitin, consistent with a decreased proteasomal activity.

### Effects of 15d-PGJ_2_ on Cell Signal-Induced Degradation of IκB-α and p105 and on NF-κB Nuclear Translocation

**Figure 3:**
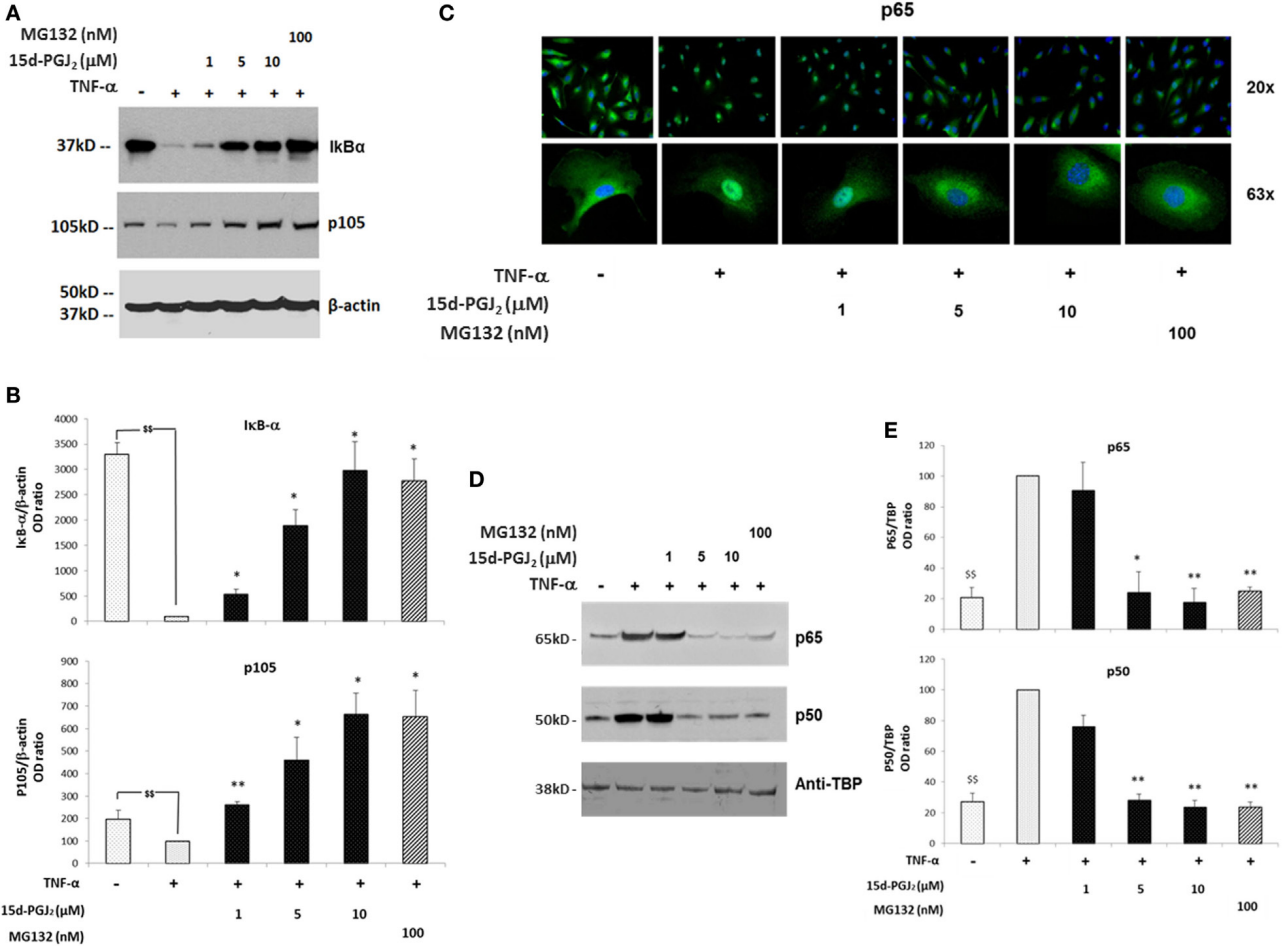
**Effects of 15d-PGJ_2_ on cell signal-induced degradation of IκB-α and p105 and on NF-κB nuclear translocation**. **(A)** EC were treated with 15d-PGJ_2_ (1–10 μM) or MG132 (100 nM) for 18 h, followed by 30min stimulation with TNF-α (0.2 ng/ml). Proteasomal IkB-α degradation and p105 processing were estimated by Western blotting of the indicated protein, and β-actin was used as loading control. The effect of the proteasome inhibitor MG132 is also shown. The data for three independent experiments are shown. **(B)** Protein levels (assessed as ratio of protein OD normalized to the OD of β-actin) are shown as percentage of control (vehicle + TNF-α). Data are expressed as mean ± SEM of three independent experiments. ^$$^(*p* < 0.01) vehicle vs. TNF-α; **p < 0.01 and *p < 0.05 15d-PGJ_2_ or MG132 vs. TNF-α. **(C)** Epifluorescence microscopy was performed on EC treated with 15d-PGJ_2_ (1–10 μM) or MG132 (100 nM) for 18 h prior to activation with TNF-α (0.2 ng/ml) for 30 min at 20× (top panel) and 63× (bottom panel) magnification. DNA was stained with DAPI dye (blue), and NF-κB was stained with an anti p65 antibody and Alexa-488 conjugated secondary antibody (green). Images are representative of three independent experiments. **(D)** Western blot analysis of p65 and p50 levels evaluated in nuclear extracts of TNF-α-activated EC pre-incubated with or without 15d-PGJ_2_ or MG132 (100 nM). An anti-TATA-binding protein (TBP) was used as loading control for EC nuclear fractions. **(E)** Protein levels (assessed as ratio of protein OD normalized to the OD of TBP) were shown as percentage of control (vehicle + TNF- α) (*n* = 3); **p < 0.01 and *p < 0.05 15d-PGJ_2_ or MG132 vs. TNF-α.

The proteasome system plays a critical role in the regulation of the inflammatory process in part by modulating the activation of the NF-κB pathway. In particular, the proteasome regulates two crucial steps of NF-κB activation, IκB-α degradation, and p105 processing, leading to the formation of active p50/p65 dimers. If proteasome activity is inhibited by 15d-PGJ_2_, then degradation of both IkB-α and p105 would be prevented, and consequently, NF-κB pathway activation would be suppressed. As shown in Figures 3A,B, treatment of EC with increasing concentrations of 15d-PGJ_2_ (1–10 μM) or with the proteasome inhibitor MG132 prevented the proteolysis of IκB-α and p105 induced by TNF-α. It should be noted however that 15d-PGJ_2_ inhibits a number of other components of the NF-κB signaling pathway resulting in the accumulation of IκB-α, including IKK and the p50 subunit, the latter by direct covalent modification (19).

In addition, NF-κB is normally held in an inactive state in the cytoplasm through interactions with IκB-α. TNF-α induces IκB-α degradation, freeing NF-κB to translocate to the nucleus and initiate transcription of proinflammatory genes. The nuclear localization of active NF-κB was detected by immunofluorescence experiments using a fluorescein isothiocyanate (FITC)-conjugated p65 antibody (Figure 3C). While in resting EC, p65 showed strong staining in an exclusively cytoplasmic localization, p65 was predominantly localized to the nucleus in the presence of TNF-α. In contrast, in the presence of 15d-PGJ_2_ and MG132, nuclear translocation of p65 induced by TNF-α was reduced. These findings were confirmed by Western blotting of nuclear fractions obtained from TNF-α-activated EC, which showed that 15d-PGJ_2_ inhibited the nuclear localization of p65 and p50 induced by TNF-α, as did the synthetic proteasome inhibitor, MG132 (Figures 3D,E). In summary, in TNF-α-activated EC, treatment with 15d-PGJ_2_ resulted in the inhibition of the NF-κB pathway activation, by modulating the processing the NF-κB inhibitors, similarly to the effects of the proteasome inhibitor MG132.

### Effect of 15d-PGJ_2_ on Adhesion Molecule mRNA and Protein Expression in EC

**Figure 4:**
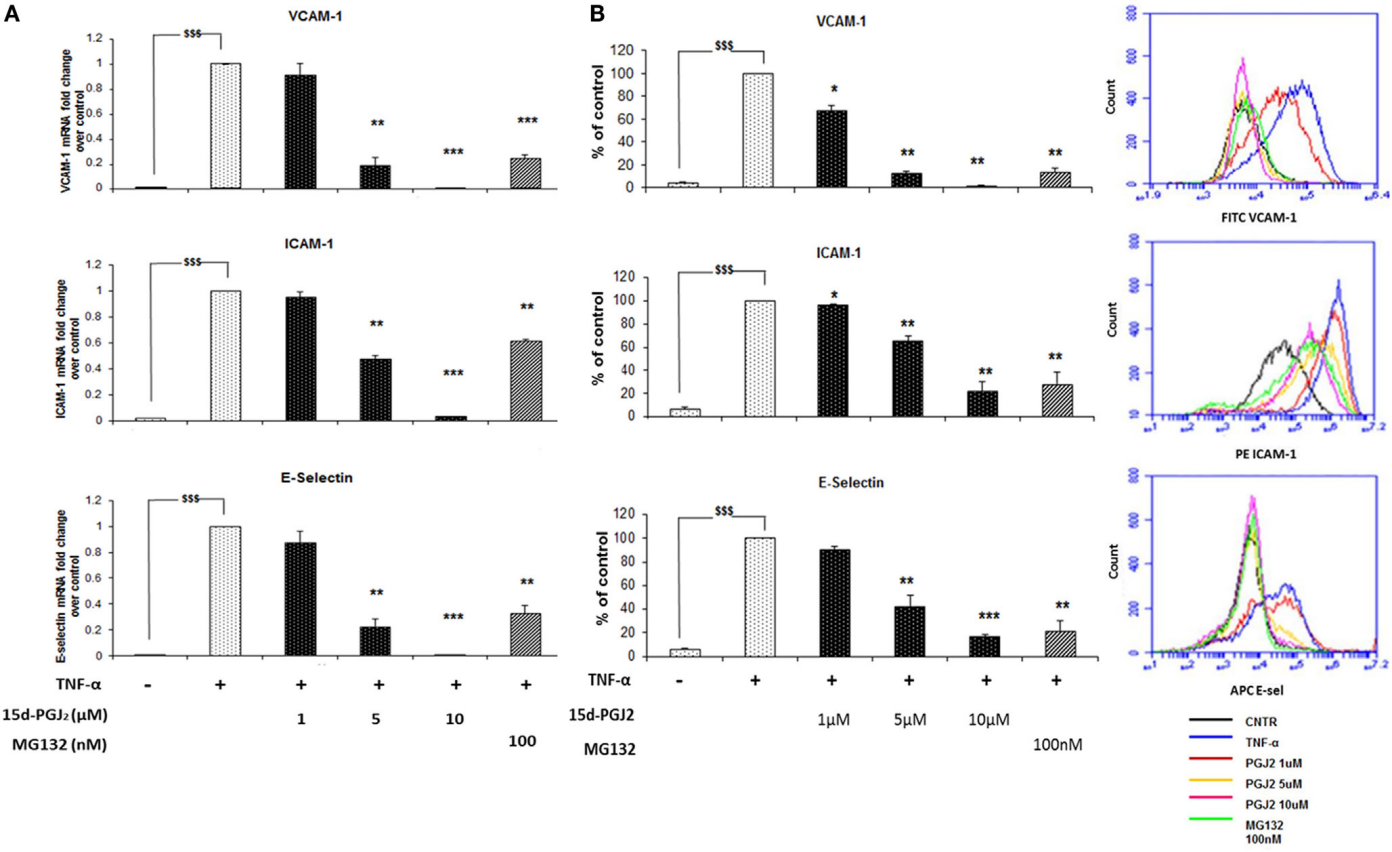
**15d-PGJ_2_ inhibits mRNA expression of adhesion molecules**. **(A)** VCAM-1, ICAM-1, and E-selectin gene expression was performed in EC treated with 15d-PGJ_2_ (1–10 μM) or MG132 (100 nM) for 18 h, followed by 6 h stimulation with TNF-α (0.2 ng/ml). The treatment of EC with 15d-PGJ_2_ inhibited gene expression of all the adhesion molecules in a dose-dependent fashion. Similar effects were obtained in the MG132-treated samples. Data are expressed as mean ± SEM of three independent experiments. **(B)** The quantification of protein surface expression levels of the adhesion receptors VCAM-1, ICAM-1, and E-selectin was performed by flow cytometry analysis on TNF-α-activated EC (6 h, 0.2 ng/ml) pre-incubated with various concentration of 15d-PGJ_2_ or MG132 (100 nM). Results show a dose-dependent decrease of protein levels in EC treated with the 15d-PGJ_2_, as well as MG132. Data are expressed as mean ± SEM of three independent experiments. Data shown as percentage of control (vehicle + TNF-α-activated EC); ^$$$^(*p* < 0.001) vehicle vs. TNF-α; ***p < 0.001, **p < 0.01, and *p < 0.05 15d-PGJ_2_ or M132 vs. TNF-α.

Inhibition of the nuclear translocation of p65 and p50 by 15d-PGJ_2_ would be expected to suppress the expression of NF-κB target genes induced in response to TNF-α, including adhesion molecules and chemokines. The effect of 15d-PGJ_2_ on TNF-α-induced expression of adhesion molecules was determined in EC activated by TNF-α for 6 h (Figure 4A). TNF-α markedly increased mRNA expression of VCAM-1, ICAM-1, and E-selectin compared to vehicle control (*p* < 0.001). Pretreatment with either 15d-PGJ_2_ or MG132 significantly suppressed the expression of mRNA for VCAM-1, ICAM-1, and E-selectin. These results were confirmed by flow cytometry (Figure 4B), which showed a dose-dependent decrease of EC surface protein expression of VCAM-1, ICAM-1, and E-selectin.

### Effect of 15d-PGJ_2_ on Human Monocyte Adhesion to Activated Endothelial Cells

**Figure 5:**
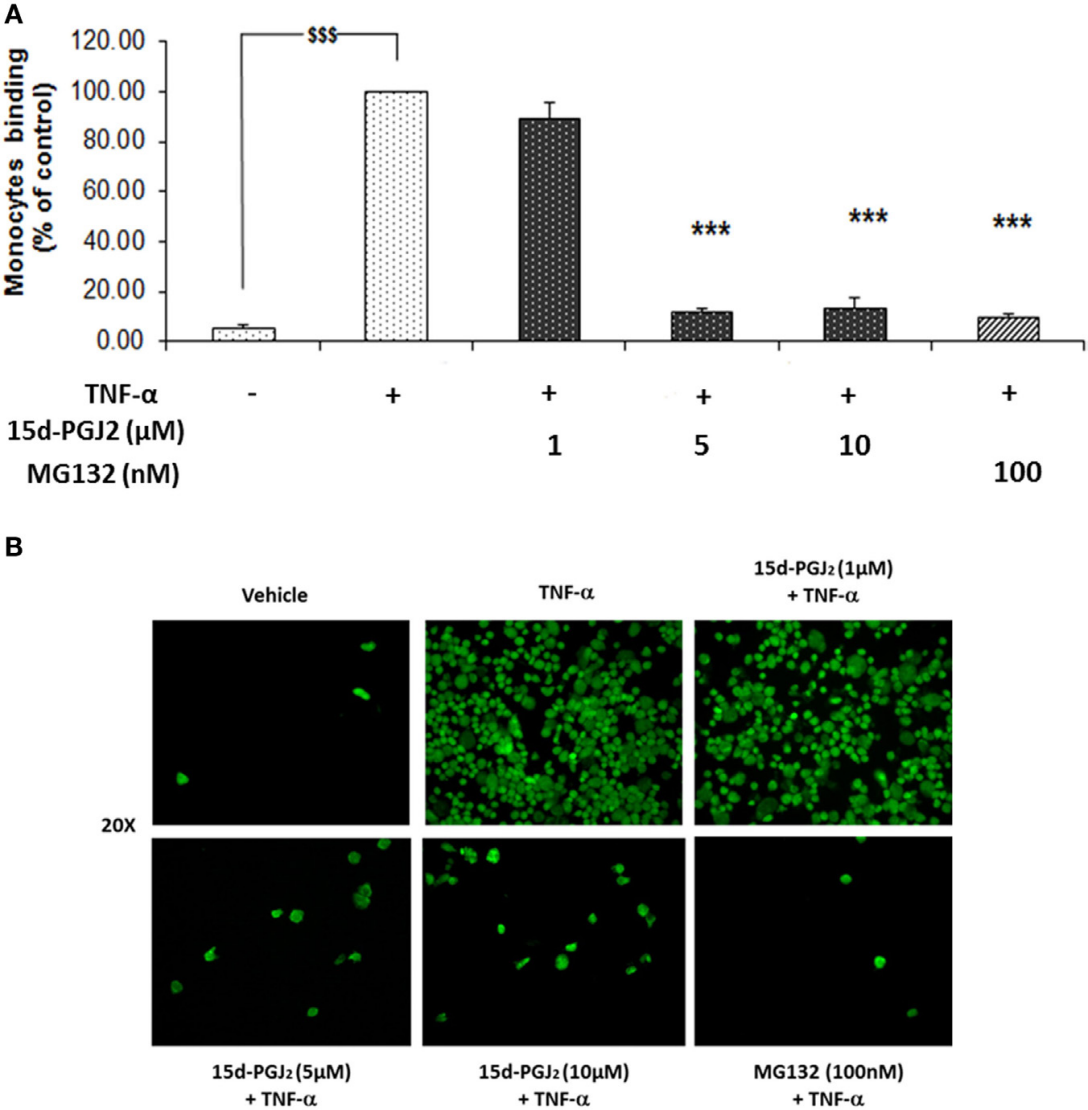
**15d-PGJ_2_ inhibits human monocyte adhesion to endothelial cells**. **(A)** EC were treated with 15d-PGJ_2_ or MG132 for 18 h, followed by 6 h stimulation with TNF-α (0.2 ng/ml), and a static adhesion assay with fluorescence-labeled THP-1 human monocytes was performed. Adherent monocytes were measured in a plate fluorescence reader with 485-nm excitation and 530-nm emission wavelengths. Data were calculated as mean ± SEM of three independent experiments. Data are reported as percentage of TNF-α-activated EC; ^$$$^(*p* < 0.001) vehicle (ethanol) vs. TNF-α; ***p < 0.001 treatments vs. TNF-α. **(B)** Representative fluorescence microscopy photomicrographs of monocyte adhesion to EC are shown.

The induction of ICAM-1, VCAM-1, and E-selectin, among others, facilitates the rolling and firm adhesion of monocytes to the EC surface of the vessel wall, a key process in vascular inflammation. Having confirmed the ability of 15d-PGJ_2_ to reduce adhesion molecule expression in EC treated with TFN-α, further experiments were undertaken to examine the effect of 15d-PGJ_2_ on monocyte adhesion to TFN-α-treated EC. As shown in Figures 5A,B, TNF-α caused a significant increase in the adhesion of human monocytes compare to vehicle (~19-fold, *p* < 0.001). Pretreatment with 15d-PGJ_2_ inhibited the adhesion of monocytes on TNF-α-activated EC (by 11 ± 6, 89 ± 2, and 87 ± 4% with 1, 5, and 10 μM 15d-PGJ_2_, respectively). Similarly, MG132 decreased adhesion of monocytes by 90 1%, consistent with a role for the proteasome in regulating the pathways leading to monocyte adhesion.

### Effect of 15d-PGJ_2_ on EC-Conditioned Medium-Induced Monocyte Migration

**Figure 6:**
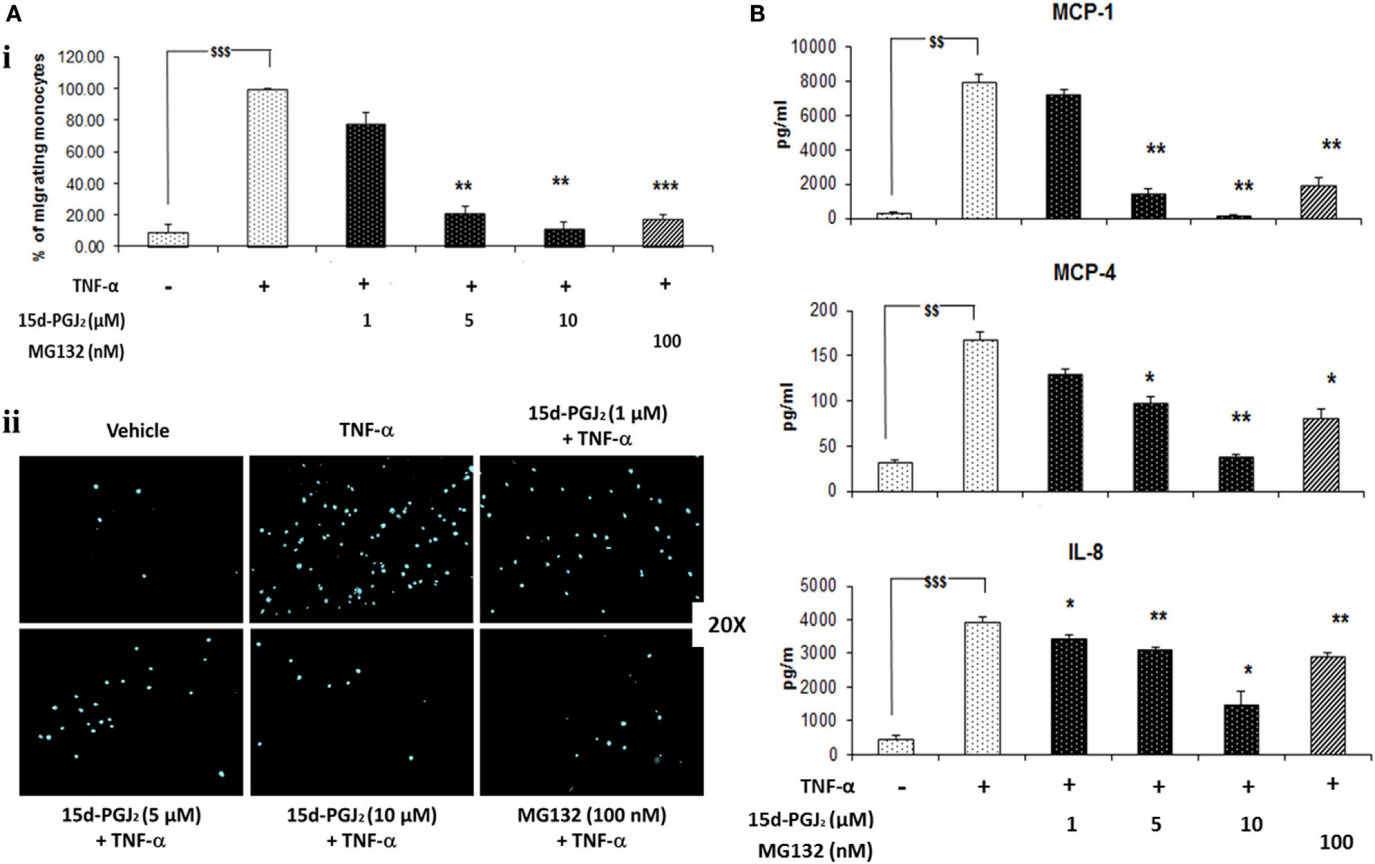
**15d-PGJ_2_ inhibits EC-conditioned-media-induced monocyte migration**. **(A)** (i) THP-1 monocyte migration in response to conditioned medium from EC that had been treated with 15d-PGJ_2_ (1–10 μM) or MG132 (100 nM) and activated with TNF-α was assayed using a 24-well transwell migration plate. Migrating cells were stained with DAPI and visualized using a fluorescent microscope. The number of cells in five random 20× fields was counted, and the average value was expressed as a percentage of TNF-α-activated EC. (ii) Representative fluorescence microscopy photomicrographs of migrating monocytes are shown. Each experiment was carried out independently three times. Data were calculated as mean ± SEM of three independent experiments and reported as percentage of TNF-α-activated EC; ^$$$^(*p* < 0.001) vehicle vs. TNF-α; ***p < 0.001 and **p < 0.01 treatments vs. TNF-α alone. **(B)** 15d-PGJ_2_ suppresses chemokine generation. Conditioned-media from EC treated with 15d-PGJ_2_ (1–10 μM) or MG132 (100 nM) and activated with TNF-α was assayed to measure IL-8, MCP-1, and MCP-4 levels by ELISA. The results are from three independent experiments. Data were calculated as mean ± SEM and reported as percentage of TNF-α-activated EC; ^$$$^(*p* < 0.001), ^$$^(*p* < 0.01) vehicle vs. TNF-α; **p < 0.01 and *p < 0.05 treatments vs. TNF-α alone.

Monocytes may be recruited to the vascular endothelium in early atherosclerosis through chemotactic factors such as MCP-1, MCP-4, and IL-8 that are released in response to activation of NF-κB pathway. As shown in Figure 6A, conditioned medium from 15d-PGJ_2_-treated EC that had been exposed to TNF-α promoted monocyte chemotaxis and this was suppressed by prior incubation of the EC with 15d-PGJ_2_ in a concentration-dependent manner. The proteasomal inhibitor MG132 showed similar effects, inhibiting moncoyte chemotaxis by the conditioned medium by 78 ± 2%. Furthermore, as shown in Figure 6B, both 15d-PGJ_2_ and MG132 significantly decreased EC generation of MCP-1, MCP-4, and IL-8 in the conditioned media assayed by ELISA, explaining, at least in part, their inhibition of monocyte chemotaxis.

## Discussion

15d-PGJ_2_ downregulates expression or activity of proinflammatory genes induced by various stimuli (e.g., TNF-α, IL-1b, LPS, etc.) in several cell and potentially acts as an endogenous suppressor of inflammation (29, 30). There are a number of possible mechanisms for this activity (31). First, 15d-PGJ_2_ is a ligand for peroxisome proliferator-activated receptor gamma (PPAR-γ), a member of the nuclear receptor superfamily and a transcription factor with pleiotropic effects on adipocyte differentiation, glucose homeostasis, lipid metabolism, cell growth, and inflammation (29, 32). Second, 15d-PGJ_2_ also exerts effects by covalently modifying proteins through the reactive α,β-unsaturated carbonyl group located in the cyclopentenone ring, a structural feature unique to J series prostaglandins (Figure 1A) (9–15). In earlier studies, we used biotin-15d-PGJ_2_ to capture a wide range of protein candidates in EC (27). The biotin binds to the carboxyl group leaving the cyclopentenone free to interact with free thiol groups of proteins. We also showed that 15d-PGJ_2_ covalently modifies proteins in multiple pathways, thereby altering their cellular functions; for example, 15d-PGJ_2_ covalently modified nuclear transport proteins, blocked nuclear transport and as a consequence altered gene expression (14).

In the present study, we further examined the set of proteins previously reported (27) and noted a disproportionate representation of proteins from the proteasome, a cellular complex that ubiquinates and degrades cellular proteins. The 13 proteins identified were solely in the 19S regulatory subunit, demonstrating extraordinary selectivity. The results were confirmed by anti-biotin Western blot for several of the 19S component proteins and two 20S protein as control. As we had deliberately used a high concentration of 15d-PGJ_2_ to capture a wide range of proteins, we next examined the interaction between biotin-15d-PGJ_2_ and purified proteasome proteins. This demonstrated that the interaction occurred at nanomolar concentrations of the lipid and could be blocked by excess unlabeled 15d-PGJ_2_. We further showed that 15d-PGJ_2_ inhibited proteasome activity in human EC, as did MG132, a known proteasome inhibitor which acts by covalently binding to the beta subunits of the proteasome. Proteasome inhibitors are either synthetic compounds or natural products and have served as tools in the discovery of UPS targets and their role in different cellular processes. Several proteasome inhibitors are currently used in the treatment of cancer (33, 34). The main consequence of proteasome inhibition is a decrease in the rate of protein degradation in cells and as a result to a rapid accumulation of proteins conjugated to ubiquitin (Figure 2C).

**Figure 7:**
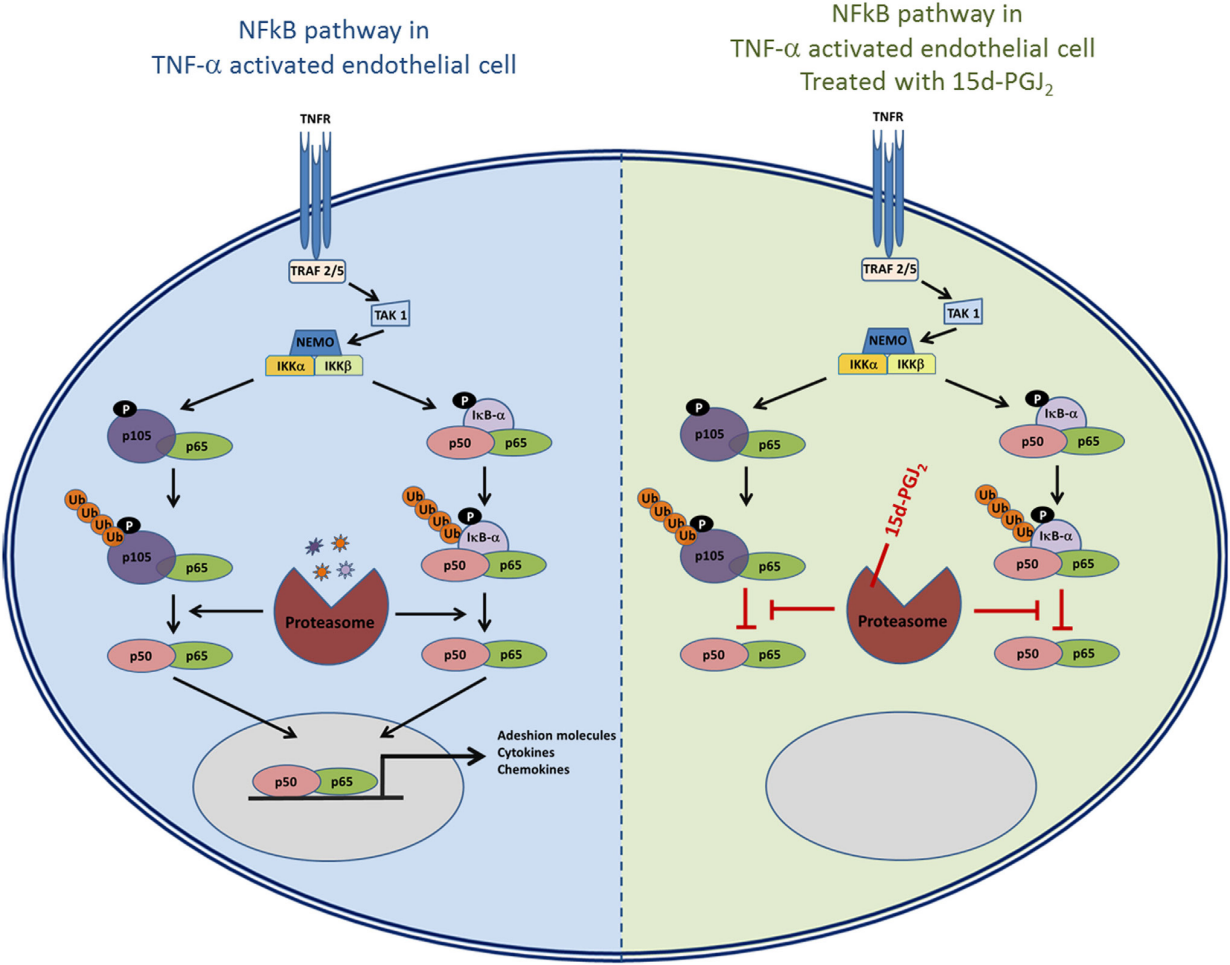
**The role of proteasome in the NF-κB pathway and the possible effects of inhibition of proteasome activity by 15d-PGJ_2_**.

As 15d-PGJ_2_ acts as an inhibitor of proteasome function, we next explored its effects on the activation of the NF-κB pathway in EC in response to TNF-α. The ubiquitous eukaryotic transcription factor NF-κB plays a central role in cellular inflammation processes (35, 36). NF-κB is sequestered in the cytoplasm within an inactive complex comprised of a family of repressors, including IκB-α. Phosphorylation of IκB-α triggers its conjugation to ubiquitin and subsequent degradation by the proteasome. As a result, NF-κB is liberated from the inhibitory protein and translocates to the nucleus where it induces the expression of genes, including genes for adhesion molecules, cytokines, and chemokines. Inhibition of the proteasome has an additional effect on the NF-κB pathway as p50 and p52 are generated from their corresponding p105 and p100 precursors by ubiquitin–proteasome-dependent degradation. Thus, the processing of IκB-α and p105 is modified by proteasome inhibitors such as MG132 (37, 38). Both 15d-PGJ_2_ and MG132 inhibited IκB-α degradation and p105 processing in response to TNF-α in our experiments. The consequence of preventing IκB-α degradation and p105 processing is to stablilise NF-κB and prevent its activation and downstream effects. Indeed, 15d-PGJ_2_ blocked p65 and p50 nuclear localization in EC in response to TNF-α and the expression of inflammatory genes including adhesion receptors (VCAM-1, ICAM-1, and E-sel) and chemokines (Figure 7).

The incomplete inhibition of proteasomal activity by 15d-PGJ_2_ (~44%, *p* < 0.01) was comparable to that observed with MG132 (~58%, *p* < 0.01). Therefore, these results explain only in part the complete inhibition of adhesion molecule expression and monocyte recruitment to activated EC. It is possible that some of the 15d-PGJ_2_ anti-inflammatory effects detected in EC reflect the modification of other proteins processed by the ubiquitin–proteasome pathway (39), interference with other steps in the NF-κB signaling pathway, or by PPAR-γ-dependent mechanisms. Given the reactivity of the cyclopentenone structure, 15d-PGJ_2_ may likewise modify other inflammatory pathways, independently of proteasome-NF-κB signaling. The proteasome system is also responsible for the regulation of transcriptional activators such as Keap1-Nrf2, P53, and JAK/STAT, which, in turn, modulate inflammatory processes, cell cycle, growth, and differentiation (40). Nrf2 plays a crucial role in cellular defense (41) and is sequestered by Keap1 in the cytoplasm, which in turn undergoes proteasomal degradation under certain stress conditions, releasing Nrf2 (42). It has also been shown that 15d-PGJ_2_ is able to activate the Nrf2 regulatory pathway by covalent modification of Keap1 (43). The JAK/STAT cascade is an additional inflammatory signaling pathway modified by 15d-PGJ_2_ (44). 15d-PGJ_2_ is also an endogenous agonist of PPAR-γ, a nuclear receptor that modulates a variety of cellular processes (8, 45). Crystallographic analysis of PPAR-γ revealed that the ligand-binding domain (LBD) covalently binds to 15d-PGJ_2_ inducing conformational changes in the loop region of LBD (46). Finally, 15d-PGJ_2_ can modulate NF-κB activity by interfering directly with members of the NF-κB pathway independently of PPAR-γ activation or proteasome inhibition. For example, 15d-PGJ_2_ inhibited IKK and the DNA binding of NF-κB in several cell types (18), and 15d-PGJ_2_ directly inhibited and modified IKK-β subunit of IKK (17).

The implication of these findings is that 15d-PGJ_2_ may act as an endogenous suppressor of diseases where inflammation is a hallmark, for example, in atherosclerosis. The UPS has been implicated in the pathogenesis of this disease (25), and 15d-PGJ_2_ has been identified in atherosclerotic plaque (47). Furthermore, NF-κB is involved in several key processes that influence the development of atherosclerotic plaque (48, 49) through the activation of genes involved in inflammation. Consistent with this, modulators of proteasome activity and NF-κB inhibition have been demonstrated to be atheroprotective (50–52).

There is a question as to whether cells can produce sufficient amounts of free 15d-PGJ_2_ to have any significant effect. Whereas measured concentrations of 15d-PGJ_2_ *in vivo* are in the picomolar to nanomolar range, the biological effects in *in vitro* experiments are seen at micromolar concentrations. One explanation for the discrepancy is that assays of 15d-PGJ_2_ *in vivo* are designed to detect the free prostaglandin, while 15d-PGJ_2_ is largely protein bound. Binding to serum proteins (53), as well as sequestration by intracellular components (54) and their export from cells as water soluble glutathione *S*-conjugates (55), could explain the requirement of micromolar concentrations of 15d-PGJ_2_ to exert biological effects when added exogenously. The experiments here showed that exposure of purified 19S particle of the proteasome to biotin-15d-PGJ_2_ resulted in a dose-dependent formation of 15d-PGJ_2_–protein complexes at nanomolar concentration of biotin-15d-PGJ_2_, demonstrating the interaction between proteasome and 15d-PGJ_2_ at levels of prostaglandin that may be achieved *in vivo*. One might also speculate that localized concentrations of cyPG in the high nanomolar range may be possible given that COX isozymes functionally couple with downstream isozymes at discrete locations, such as the nuclear membrane. Finally, as 15d-PGJ_2_ exerts its biological effects through irreversible modification of cellular proteins, 15d-PGJ_2_ could induce a cumulative inactivation of protein targets over time even at very low rates of production.

In conclusion, this work was designed to identify novel protein targets of 15d-PGJ_2_ that may be responsible for its anti-inflammatory effects. We found that 15d-PGJ_2_ covalently modifies components of the proteasome thereby inhibiting its functions. The results raise the possibility that 15d-PGJ_2_ may regulate inflammatory processes in EC by modulating proteasome activity. Further understanding of the mechanisms involved may identify novel targets for the development of anti-inflammatory drugs.

## Author Contributions

SM participated in the design of the study, carried out the experiments, and prepared the manuscript. PE participated in the design of the study. DF conceived and designed the study and prepared the manuscript. All the authors read and approved the final manuscript.

## Conflict of Interest Statement

The authors declare that the research was conducted in the absence of any commercial or financial relationships that could be construed as a potential conflict of interest.

## Footnotes

^1^www.pantherdb.org.

^2^www.ebi.ac.uk/pride.
